# P-518. High Acceptance of Integrating HIV Pre-Exposure Prophylaxis into Gender Affirming Care among Transpeople Receiving Telemedicine Gender Affirming Hormone Therapy across Rural, Suburban and Urban Areas in the United States

**DOI:** 10.1093/ofid/ofae631.717

**Published:** 2025-01-29

**Authors:** Nancy Aitcheson, Caroline O’Brien, Yixuan Feng, Yun Li, Moira Kyweluk, Florence Momplaisir, Nadia Dowshen

**Affiliations:** University of Pennsylvania, Atlanta, Georgia; Perelman School of Medicine, Philadelphia, Pennsylvania; The Children's Hospital of Philadelphia, Durham, North Carolina; University of Pennsylvania, Atlanta, Georgia; Plume Health, Philadelphia, Pennsylvania; University of Pennsylvania, Atlanta, Georgia; Children’s Hospital of Philadelphia, Philadelphia, Pennsylvania

## Abstract

**Background:**

Transgender people are disproportionately impacted by the HIV epidemic, experiencing heightened risk factors for seroconversion and barriers to HIV prevention, such as a dearth of gender-affirming medical providers and concerns about drug-drug interactions with existing hormone regimens. Recent literature indicates urban-residing transgender women with access to gender affirming care (GAHC) may have higher awareness and uptake of pre-exposure prophylaxis for HIV (PrEP). This project explores PrEP knowledge, experience, and acceptability among individuals accessing GAHC via telemedicine in urban and rural settings.

**Methods:**

We surveyed clients of a telemedicine gender affirming healthcare company in December 2023, using purposeful sampling to ensure representation of all genders, and across geographies. Participants were >18 years old and currently accessing GAHC. Zip codes were classified by urban, suburban, and rural by modified Rural Urban Commuting Score. The online survey asked about knowledge, perceived barriers, and willingness to use PrEP. Chi-squared tests were used for statistical comparisons across geographical settings.

**Results:**

Of 387 respondents, 212 identified as transfeminine, 170 as transmasculine and 5 had unknown gender identifications. Participants were spread across geographies of rural (53), suburban (126) and urban (168). Zip codes were missing for 40 participants. 83% were white and 51% were between the ages of 18-25. 24% reported not knowing their HIV status. Awareness of PrEP did not vary significantly across geographies, with 57% of rural, 56% of suburban, and 64% of urban residents reporting being aware of PrEP prior to the survey. Less than 3% had ever taken PrEP. Two-thirds (67%) of participants were willing to take PrEP if recommended and prescribed by their GAHC provider and did not differ across geographical settings.

**Conclusion:**

Compared to recent literature, this survey of geographically diverse trans people engaged in GAHC demonstrated lower PrEP awareness and use of PrEP. However, there was consistent interest in PrEP if recommended and prescribed by a GAHC provider. Integrating HIV prevention into GAHC may be an effective avenue to reaching a vulnerable community.

**Disclosures:**

**All Authors**: No reported disclosures

